# The Toxicity of a Mutant Prion Protein Is Cell-Autonomous, and Can Be Suppressed by Wild-Type Prion Protein on Adjacent Cells

**DOI:** 10.1371/journal.pone.0033472

**Published:** 2012-03-12

**Authors:** Emiliano Biasini, Jessie A. Turnbaugh, Tania Massignan, Pietro Veglianese, Gianluigi Forloni, Valentina Bonetto, Roberto Chiesa, David A. Harris

**Affiliations:** 1 Department of Biochemistry, Boston University School of Medicine, Boston, Massachusetts, United States of America; 2 Dulbecco Telethon Institute, Milan, Italy; 3 Department of Neuroscience, Mario Negri Institute, Milan, Italy; 4 Department of Biochemistry and Molecular Pharmacology, Mario Negri Institute, Milan, Italy; Nagasaki University Graduate School of Biomedical Sciences, Japan

## Abstract

Insight into the normal function of PrP^C^, and how it can be subverted to produce neurotoxic effects, is provided by PrP molecules carrying deletions encompassing the conserved central region. The most neurotoxic of these mutants, Δ105–125 (called ΔCR), produces a spontaneous neurodegenerative illness when expressed in transgenic mice, and this phenotype can be dose-dependently suppressed by co-expression of wild-type PrP. Whether the toxic activity of ΔCR PrP and the protective activity or wild-type PrP are cell-autonomous, or can be exerted on neighboring cells, is unknown. To investigate this question, we have utilized co-cultures of differentiated neural stem cells derived from mice expressing ΔCR or wild-type PrP. Cells from the two kinds of mice, which are marked by the presence or absence of GFP, are differentiated together to yield neurons, astrocytes, and oligodendrocytes. As a surrogate read-out of ΔCR PrP toxicity, we assayed sensitivity of the cells to the cationic antibiotic, Zeocin. In a previous study, we reported that cells expressing ΔCR PrP are hypersensitive to the toxic effects of several cationic antibiotics, an effect that is suppressed by co-expression of wild type PrP, similar to the rescue of the neurodegenerative phenotype observed in transgenic mice. Using this system, we find that while ΔCR-dependent toxicity is cell-autonomous, the rescuing activity of wild-type PrP can be exerted *in trans* from nearby cells. These results provide important insights into how ΔCR PrP subverts a normal physiological function of PrP^C^, and the cellular mechanisms underlying the rescuing process.

## Introduction

Prion diseases are fatal neurodegenerative disorders of humans and animals that are characterized by dementia, motor dysfunction, cerebral amyloidosis, and spongiform degeneration of the brain [1]. The diseases manifest themselves in genetic, infectious, and sporadic forms. All of these forms are caused by conformational conversion of PrP^C^, a normal, cell surface glycoprotein into a β-sheet-rich, aggregated isoform termed PrP^Sc^. A great deal of evidence suggests that PrP^Sc^ is an infectious agent that propagates itself by seeding misfolding of PrP^C^ substrate molecules in a template-directed fashion [2], [3].

Although it is commonly accepted that PrP^Sc^ is a hallmark of prion diseases, emerging evidence suggests that its neurotoxicity relies on the presence of functional PrP^C^ molecules at the cell surface [4]. This conclusion is supported by the observation that the depletion of neuronal PrP^C^ in mice with an established prion infection reversed both neuronal loss and the progression of clinical signs, despite the continuous production of PrP^Sc^ by surrounding glial cells [5], [6]. This and other lines of evidence have sparked renewed efforts to understand the normal function of PrP^C^ and how it may be hijacked to generate toxicity [7], [8]. Multiple functions have been attributed to PrP^C^ in the last decade, including roles in cell adhesion, metal ion homeostasis, neuroprotection from various cellular stresses, and transduction of toxic signals delivered by several misfolded proteins [9], [10]. However, the physiological role of PrP^C^ remains unclear, and thus far no robust assays have been developed for testing its function *in vitro*.

A possible clue into the physiological activity of PrP^C^ is provided by transgenic (Tg) mice expressing PrP forms carrying deletions within the central region. One of these lines, expressing a mutant PrP molecule deleted for residues 105–125 (referred to as ΔCR), displays a severe neurodegenerative phenotype characterized by progressive loss of cerebellar granular neurons (CGNs), astrogliosis, and white matter vacuolization [11]. Surprisingly, ΔCR PrP, like its wild-type (WT) counterpart, is monomeric and localized mainly in lipid rafts at the cell surface, suggesting that the deletion of residues 105–125 alters a normal, physiological function of PrP, rather than causing the protein to adopt a PrP^Sc^-like conformation [12]. In addition, both neurodegeneration and clinical symptoms in Tg(ΔCR) mice are abrogated by co-expression of WT PrP, suggesting that WT and mutant molecules could be acting through the same molecular pathway, or competing for a common ligand [11]. In either case, understanding how ΔCR PrP exerts its toxic effect will likely provide important clues to the physiological activity of PrP^C^.

In previous work, we found that expression of ΔCR PrP induced a strong ion channel activity in transfected cells that could be detected by patch-clamping techniques [13], [14]. In addition, this mutant rendered a variety of cell lines, including HEK293, N2a, Sf9 and differentiated mouse neural stem cells (NSCs), hypersensitive to several cationic antibiotics, including G418, Zeocin and hygromycin [15]. This phenotype provided the basis for a novel cell culture assay to study the activity of PrP mutants, referred to as the Drug-Based Cell Assay (DBCA) [16]. Analogous to its ability to reverse the neurodegenerative phenotype of Tg(ΔCR) mice, WT PrP suppressed ΔCR PrP-induced drug hypersensitivity and ion channel activity, suggesting that similar cellular mechanisms are operative in each of these settings. Therefore, the DBCA provides an unprecedented opportunity to dissect PrP^C^ function at a cellular level.

Previous experiments establish that ΔCR PrP is toxic to neurons, and this effect is suppressed by the co-expression of WT PrP. However, it is not known whether the toxic activity of ΔCR PrP and the protective activity of WT PrP are cell-autonomous, or can be exerted on neighboring cells. Since PrP is a cell-surface protein, it could potentially interact via both kinds of mechanisms. Here, we established a novel NSC-based version of the DBCA to define whether or not the antibiotic-hypersensitizing effect of ΔCR PrP and the rescuing activity of WT PrP are cell-autonomous. We describe the intriguing observation that ΔCR PrP induces antibiotic hypersensitivity in a cell-autonomous fashion, and that this effect can be rescued by WT PrP expressed on neighboring cells. These results provide new insights into the cellular mechanisms underlying the neurotoxic and neuroprotective functions of PrP^C^.

## Results

### Characterization of mouse NSC cultures

**Figure 1:**
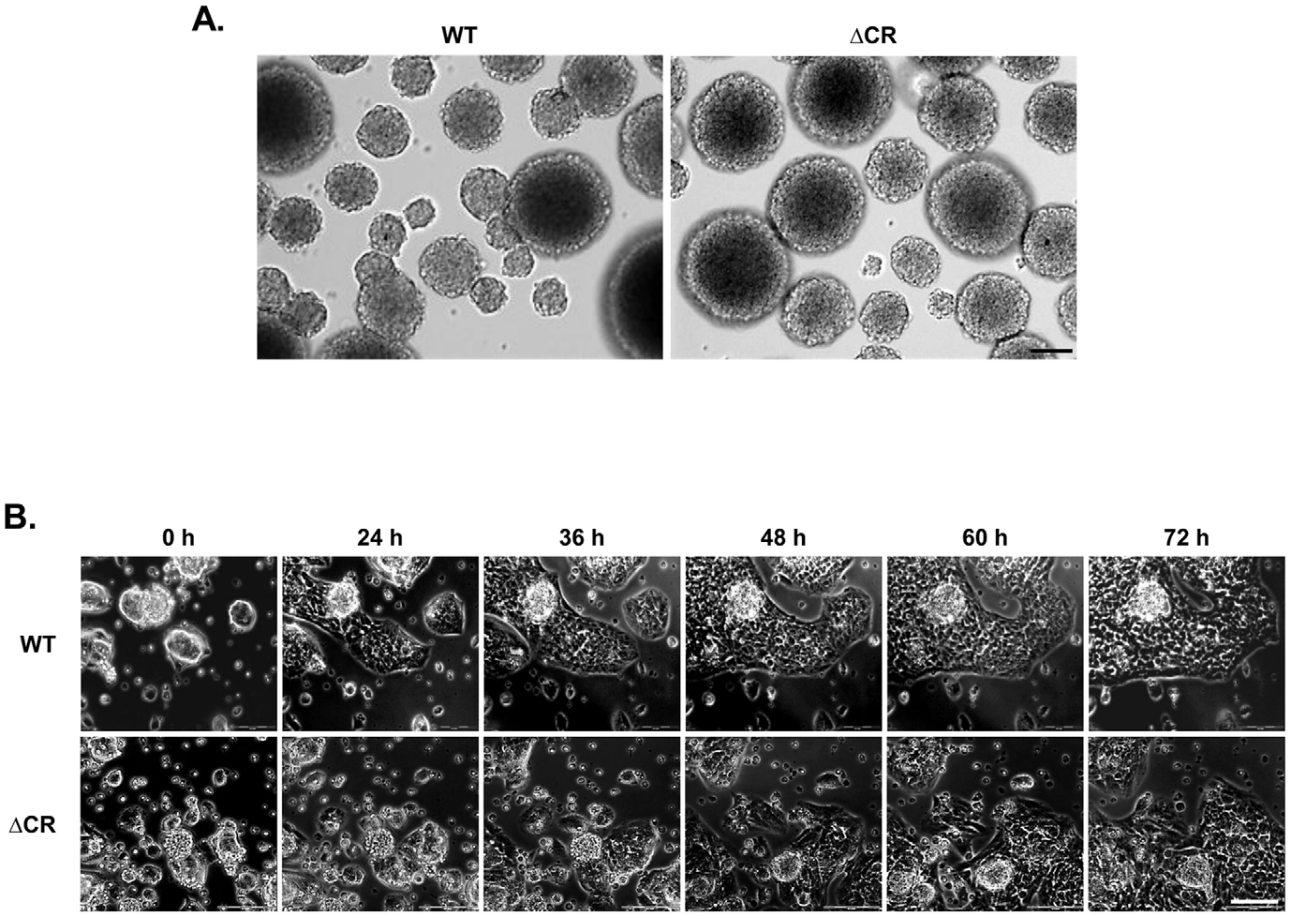
NSCs propagate as spheroid bodies and form a monolayer upon differentiation. (A) NSCs cultured in complete media (containing EGF) grow as 100–200 µm neurospheres, capable of self-renewal. (B) Phase-contrast, time-lapse microscopy shows that NSCs cultured in the absence of EGF, with the addition of serum and retinoic acid, form a monolayer within 72 h. Representative pictures from WT and ΔCR NSCs are shown. Scale bars = 100 µm.

In a preliminary set of experiments, we sought to characterize the properties of NSCs derived from several mouse lines, including non-transgenic *Prn-p^+/+^* (WT), *Prn-p^−/−^* (KO), Tg*a*20^+/+^/*Prn-p^0/0^* (Tg*a*) and Tg(ΔCR)/*Prn-p^0/0^*(ΔCR) mice. In the presence of epidermal growth factor (EGF), NSCs from all mouse lines formed spheroid bodies (or neurospheres), measuring 50–200 µm in diameter, which could replicate in culture for multiple passages (Figure 1A and data not shown). Differentiation was achieved by removing EGF between passage 2 and 5, adding serum and retinoic acid (RA), and plating the neurospheres on an adhesive substrate (poly-D-lysine/laminin). The morphological changes accompanying the differentiation of neurospheres were monitored by time-lapse microscopy for 3 days (Figure 1B). We observed that newly differentiated NSCs migrated hundreds of micrometers away from the original neurosphere, giving rise to a monolayer of cells. No obvious difference was seen between NSCs from different mouse lines.

**Figure 2:**
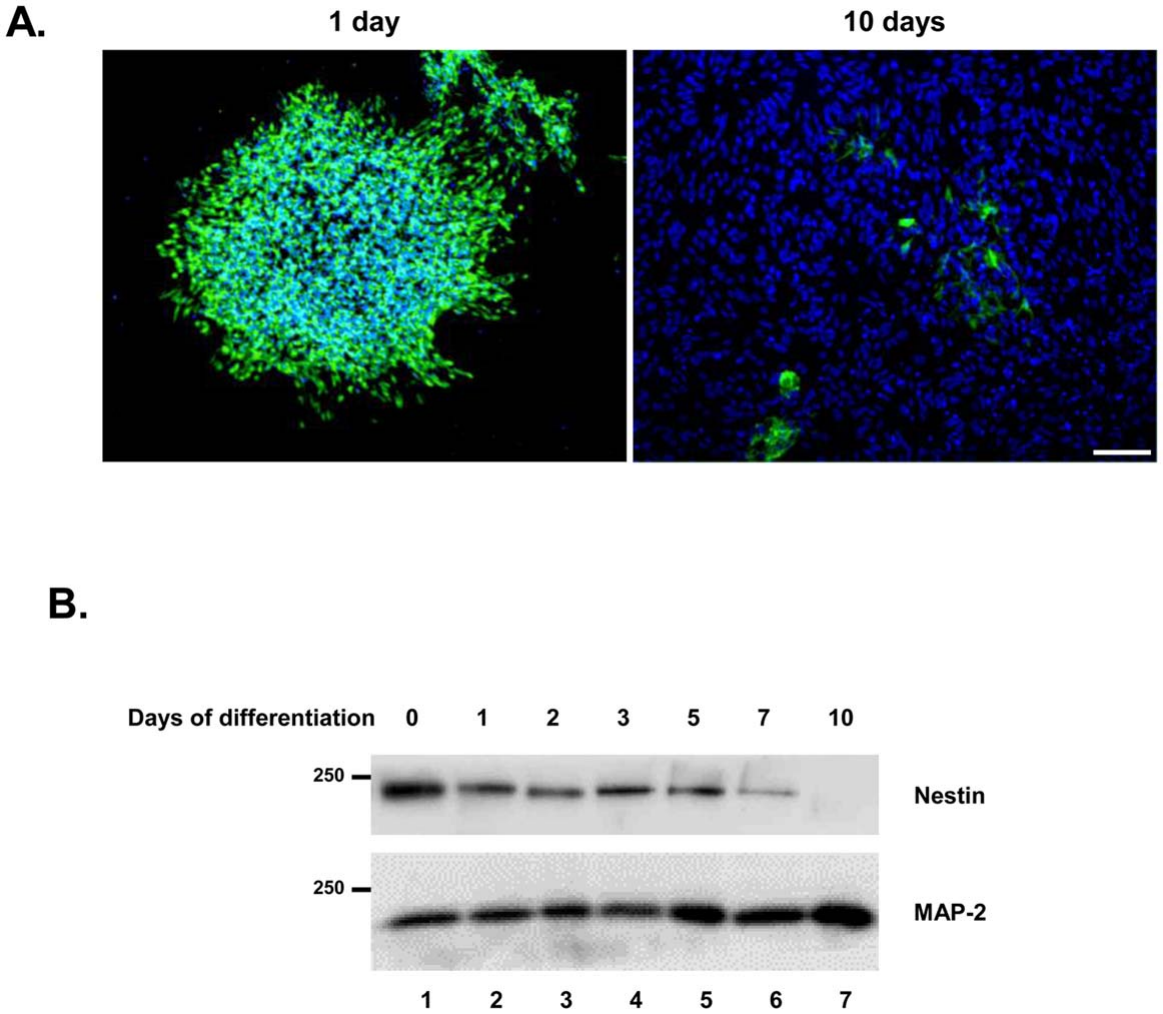
Nestin expression is decreased in differentiated NSCs. (A) NSCs grown in complete media have high levels of nestin expression (green), which decreased after 10 days in differentiation media. DAPI staining is shown in blue. Representative pictures from ΔCR NSCs are shown. Scale bar = 50 µm. (B) NSCs harvested after differentiation for the indicated number of days were immunoblotted with anti-nestin and anti-MAP-2 antibodies. After 10 days of differentiation, nestin is almost undetectable, coincident with a slight increase in MAP-2 expression. Molecular size markers are given in kDa.

In order to confirm differentiation of NSCs in culture, we measured expression of the stem cell marker, nestin, an intermediate filament protein expressed in the CNS during early stages of development [17], [18]. As expected, we observed progressive down-regulation of nestin during differentiation of ΔCR NSCs, both by immunofluorescence (Figure 2A) and Western blotting (Figure 2B). Nestin expression became almost undetectable after 10 days of differentiation, coincident with a slight increase in the neurofilament protein MAP-2 (Figure 2B). No differences were found among the different NSC lines (not shown).

**Figure 3:**
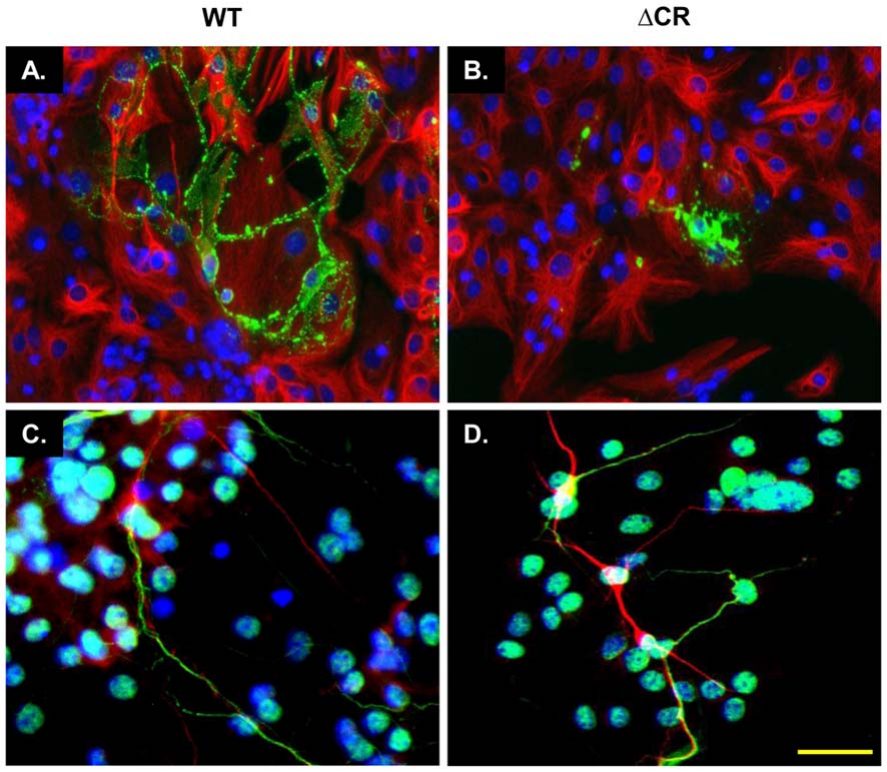
Differentiated NSCs express markers for astrocytes, oligodendrocytes, and neurons. (**A–B**) Differentiation of NSCs gives rise to cells expressing the astrocytic marker GFAP (red) and the oligodendrocyte marker MBP (green). (**C–D**) Differentiated NSC cultures also include neurons expressing dendritic (MAP-2, red) and axonal (SMI-31, green) markers. The absence of co-localization of the two neuronal markers indicates polarization of neurons after differentiation. For both (A) and (B), DAPI staining is shown in blue. Representative pictures from WT and ΔCR NSCs are shown. Scale bars = 20 µm.

Upon differentiation, stem cells are expected to give rise to mixed cultures of mature astrocytes, oligodendrocytes and neurons [19]. In order to check for the presence of these cell types, we stained differentiated NSCs with antibodies against the glial fibrillary acidic protein (GFAP), a marker of astrocytes, and the myelin basic protein (MBP), a marker of oligodendrocytes (Figure 3A–B). We estimated that astrocytes represented ∼70% of the cells in differentiated WT and ΔCR NSC cultures, while oligodendrocytes were approximately 5%. Neurons were detected by double staining with the dendritic marker MAP-2 and the axonal marker SMI-31 (Figure 3C–D). Approximately 25% of cells were positive for both markers. Importantly, we observed a lack of co-localization between the axonal marker SMI-31 and the dendritic marker MAP-2, which suggests that NSC-derived neurons are correctly polarized (Figure 3 C–D). NSCs from different mouse lines did not differ in their ability to differentiate.

**Figure 4:**
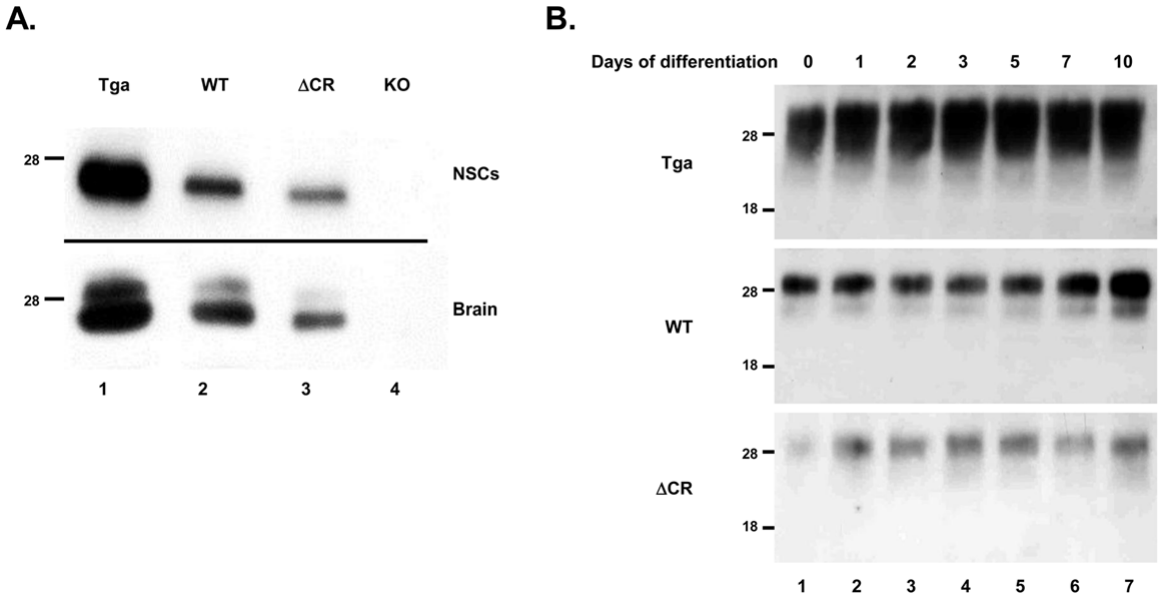
PrP expression in NSCs correlates with levels in brain and increases during differentiation. (**A**) Equivalent amounts of total protein extracts from brain homogenates or undifferentiated NSCs of the indicated genotypes were treated with PNGase F, and PrP was detected by Western blotting using 6D11 antibody. (**B**) NSCs of the indicated genotypes were collected at the indicated time points during the course of differentiation, and PrP was detected by Western blotting after treatment with PNGase. The amount of PrP increased slightly by day 10.

To verify that the level of PrP expression in differentiated NSC lines was similar to that found in the brain of the corresponding mouse line, protein extracts from undifferentiated NSCs and mouse brains were incubated with PNGase F to remove N-linked glycans, and were analyzed by Western blotting. Similar to the case in brain tissue (Figure 4A, lanes 1–3, lower panel), there was much higher PrP expression in NSCs derived from Tg*a* mice as compared to those from WT mice, with ΔCR NSCs displaying slightly lower expression than WT NSCs (Figure 4A, lanes 1–3 top panel). As expected, KO NSCs showed no PrP expression (Figure 4A, lane 4). No differences were seen between PrP expression levels in NSCs and mouse brains (Figure 4A, compare upper and lower panels). To study the expression of PrP upon differentiation, NSCs were collected at different time points and PrP expression was analyzed by Western blotting (Figure 4B). We observed a small increase in PrP expression over the 10 days of differentiation in all of the NSC lines (Figure 4B).

**Figure 5:**
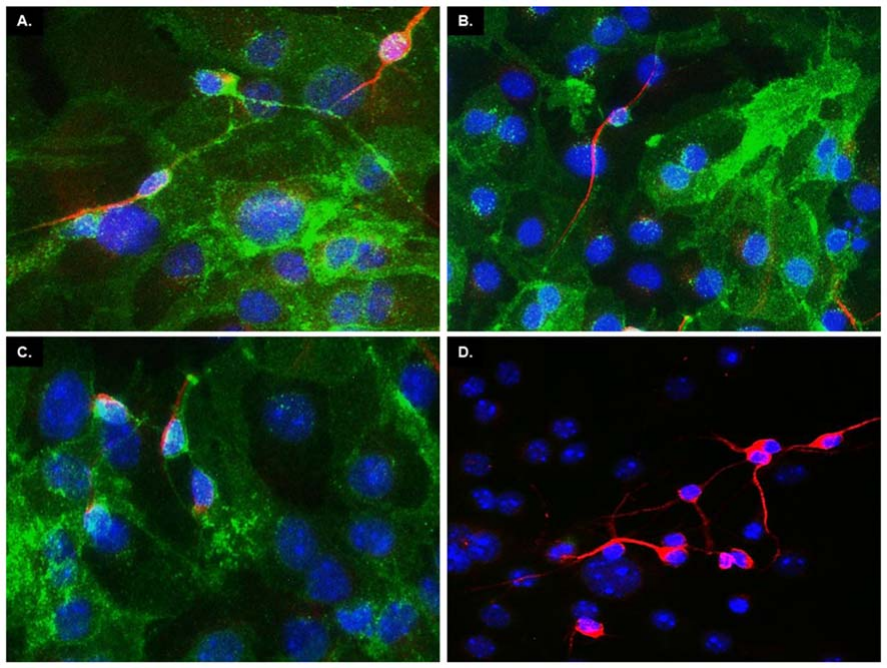
PrP is expressed on the plasma membrane in differentiated NSCs. Surface staining of differentiated NSCs shows PrP (green) expression on the surface of both neuronal (MAP-2 positive cells [red]) and non-neuronal (MAP-2 negative) cells from Tg*a* (A), WT (B), and ΔCR(C), but not KO (D) cells. PrP was detected with 6D11 antibody.

Finally, in order to visualize PrP expression in fully differentiated neuronal and non-neuronal NSCs, we co-stained cells with anti-PrP (6D11) and anti-MAP-2 antibodies. PrP was detected on the cell membrane in both neuronal (MAP-2 positive) and non-neuronal (MAP-2 negative) cells (Figure 5, A–C), while it was not detected in KO cells (Figure 5D).

These results indicate that viable NSCs can be recovered from WT, Tg*a*, KO and ΔCR mice. No difference in self-renewal or time course of differentiation was detected among the different NSC lines. Moreover, these cells correctly expressed PrP at the cell surface, with relative PrP levels identical to those observed in brain tissue from the corresponding mouse lines.

### ΔCR PrP-related hypersensitivity to Zeocin is cell autonomous

As described in a previous report, despite the potent neurotoxicity of ΔCR PrP *in vivo*, only a small percentage of dying cells (as assayed by TUNEL staining) can be detected in differentiated ΔCR NSCs, compared to controls [15]. However, the percentage of TUNEL-positive ΔCR NSCs (but not WT NSCs) dramatically increases after treatment with Zeocin, G418 or hygromicin [15]. Taking advantage of this cellular phenotype, we designed an experiment to characterize whether the drug-hypersensitizing effect of ΔCR is cell-autonomous (*in cis*) or can also be exerted *in trans* on neighboring cells.

**Figure 6:**
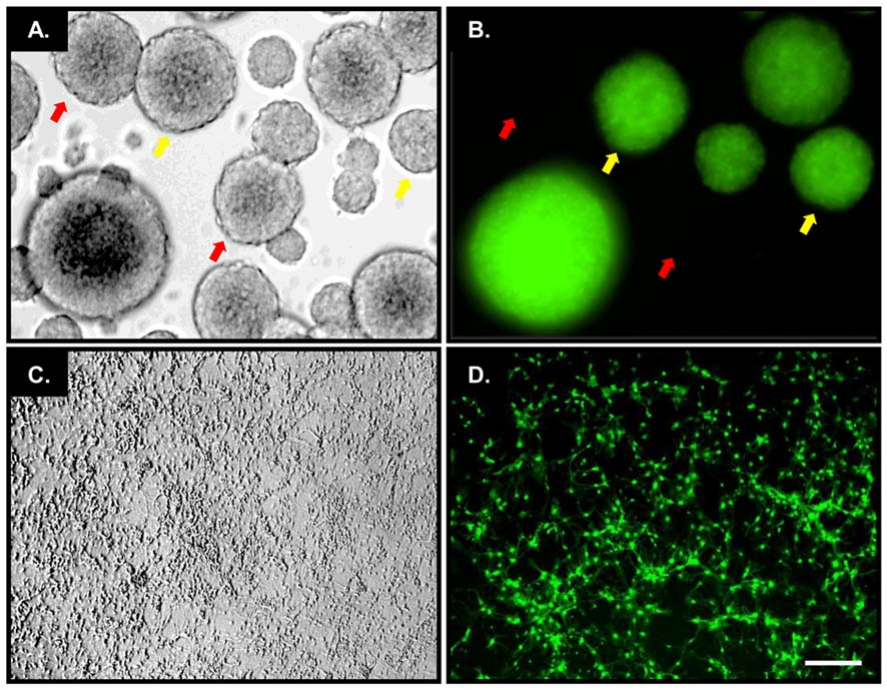
Detection of GFP-positive NSCs. (**A**) Phase contrast image of undifferentiated neurospheres of mixed GFP-positive and GFP-negative origin. (**B**) Green fluorescent signal from the same neurospheres shown in (A). Note the presence of both GFP-positive (red arrows) and GFP-negative(yellow arrows) NSCs. (**C**) Phase image of the mixed culture after differentiation. (**D**) Green fluorescent signal from the NSCs shown in (C) demonstrates that differentiated GFP-positive and GFP-negative NSCs have mixed during migration. Scale bar = 100 µm.

**Figure 7:**
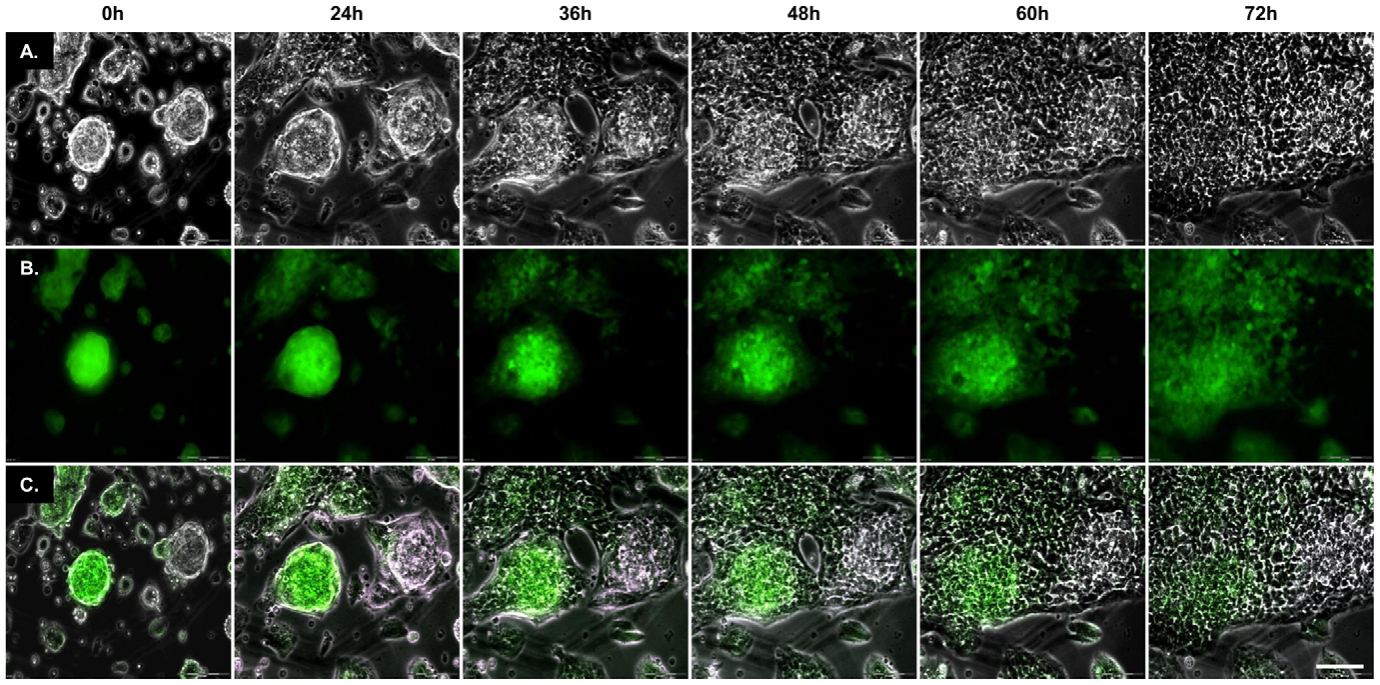
Time-lapse analysis of NSCs mixing upon differentiation. The intermixing of GFP-positive and GFP-negative NSCs during differentiation was monitored by time-lapse microscopy. Images of the differentiating cells were taken by phase contrast microscopy (**A**), or by fluorescence microscopy to detect the green fluorescent signal (**B**). A merge of the two series of images (**C**) illustrates how differentiated GFP-positive and GFP-negative migrate and mix during the first 72 h of differentiation. Representative pictures from ΔCR NSCs are shown. Scale bars = 100 µm.

KO, Tg*a* and ΔCR mice were bred with PrP-ablated Tg mice expressing a cytoplasmic form of the green fluorescent protein (GFP) under the control of the actin B promoter [referred to as Tg(*ACTB-EGFP/Prn-p^0/0^*) mice]. Mice derived from these crosses were used to prepare either GFP-positive (green) or GFP-negative (black) NSCs of each PrP genotype. To verify that we were able to discriminate between green and black NSCs in mixed cultures, black and green ΔCR NSCs were cultured together and imaged by phase contrast (Figure 6, panels A and C) or fluorescence (panels B and D) microscopy. The analysis was performed either before (Figure 6, panels A and B) or after (panles C and D) differentiation. We found that black and green cells could be easily identified both before and after differentiation. Time-lapse microscopy of differentiating black and green NSCs, imaged by phase contrast (Figure 7A) and GFP fluorescence (Figure 7B), revealed that black and green NSCs start to disperse into each other within three days of differentiation (Figure 7C). These results demonstrate that pre-mixed black and green NSCs form a uniform cell layer during differentiation, and that individual black and green cells can be identified by the presence or absence of the GFP fluorescence.

In order to test whether the presence of GFP or the mixing procedure affected the distribution of cell types, we stained co-cultures of green WT and black KO, or green ΔCR and black KO NSCs with markers for astrocytes (GFAP), neurons (MAP-2), or oligodendrocytes (MBP) (data not shown). We confirmed that the relative percentage of the different cell types was overall unchanged in the different co-coltures (astrocytes were 70%, neurons ∼25% and oligodendrocytes <5%). We also detected PrP by immunostaining in the same mixed cultures and found that the expression of PrP, either WT or ΔCR, was unchanged compared to unmixed cultures (data not shown). These results confirmed that neither the presence of GFP, nor the co-culturing method, altered the differentiation of NSCs or the expression of PrP.

To test whether ΔCR PrP exerts its effect *in cis* or *in trans*, we assayed hypersensitivity to Zeocin of mixed cultures of green KO and black ΔCR NSCs, or black KO and green ΔCR NSCs. If ΔCR PrP acts *in trans*, then both KO and ΔCR NSCs should become hypersensitive to Zeocin. In contrast, if the toxic activity of this mutant PrP is cell-autonomous, only ΔCR-expressing NSCs should be hypersensitive to Zeocin. Black and green neurospheres were mixed and differentiated for 10 days, then treated with Zeocin to unmask the ΔCR-dependent hypersensitivity to this antibiotic. Twenty-four hours after treatment, cells were stained by TUNEL to identify apoptotic cells, and reacted with DAPI to visualize the nuclei of all cells. TUNEL-positive cells were identified as belonging to either the green or black cell population, and expressed as percentage of total cells identified by DAPI staining.

**Figure 8:**
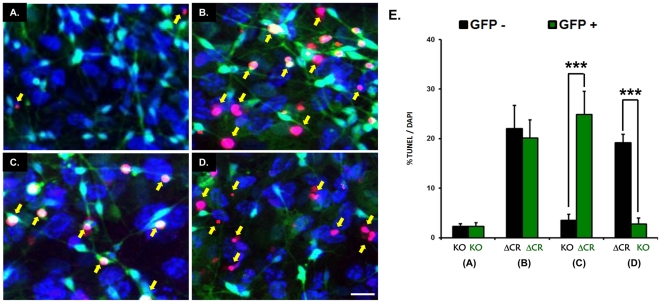
ΔCR PrP-dependent hypersensitivity to drugs is cell-autonomous. NSCs from E13.5 mouse embryos were cultured as neurospheres and differentiated for 10 days in presence of retinoic acid. Differentiated NSCs were treated for 24 hrs with Zeocin (500 µg/ml), then stained by TUNEL (red) to reveal fragmented DNA (indicated by yellow arrows) and with DAPI (blue) to stain nuclei. GFP-negative or GFP-positive NSCs from of KO or ΔCR mice were mixed as follow: (**A**) GFP-negative and GFP-positive KO cells; (**B**) GFP-negative and GFP-positive ΔCR cells; (**C**) GFP-negative KO cells mixed with GFP-positive ΔCR cells; (**D**) GFP-positive KO cells mixed with GFP-negative ΔCR cells. (**E**) The bar graph shows the number of TUNEL-positive cells, expressed as a percentage of the number of DAPI-stained cells, as determined in 5–7 fields for each sample group. Bars show means ± SEM (n = 5 independent experiments). The number of TUNEL-positive ΔCR cells, either GFP-positive or GFP-negative, was significantly higher than the number of TUNEL-positive KO cells (*** p<0.001) when ΔCR and WT cells were cultured together (groups C and D).

First, we mixed green and black KO (Figure 8A), and green and black ΔCR NSCs (Figure 8B). As expected, we found that only a small percentage (<3%) of KO cells, either green or black, were TUNEL-positive after exposure to Zeocin, while a much higher percentage (∼20%) of both green and black ΔCR cells showed sensitivity to this antibiotic (Figure 8E). These results confirmed that the presence of GFP does not interfere with the ΔCR-dependent hypersensitivity to Zeocin. Next, we mixed black KO and green ΔCR cells. This time we found that TUNEL-positive nuclei belonged almost entirely to green ΔCR cells (Figure 8C and E). This result was also confirmed in the complementary experiment, in which green KO cells were mixed with black ΔCR cells. In this case, TUNEL-positive nuclei were detected almost entirely in black ΔCR cells and not in the green KO cells (Figure 8D and E). We concluded that ΔCR PrP does not induce hypersensitivity to Zeocin in neighboring KO cells. Therefore, this PrP mutant exerts its toxic activity in a cell-autonomous fashion (*in cis*).

### WT PrP rescuing activity can be exerted *in trans*

We previously demonstrated that the ΔCR-dependent hypersensitivity to Zeocin could be rescued by co-expression of WT PrP in the same cells (*in cis*) [15]. By using the experimental paradigm described above, we tested whether such rescuing activity of WT PrP could also be exerted by expression of WT PrP on neighboring cells (*in trans*). Cultures of green or black Tg*a* and ΔCR NSCs were mixed, differentiated and treated with Zeocin. If WT PrP exerts its rescue effect *in trans*, then both Tg*a* and ΔCR NSCs should be resistant to Zeocin. Conversely, if the rescuing activity is cell-autonomous, then only Tg*a* NSCs should be protected.

**Figure 9:**
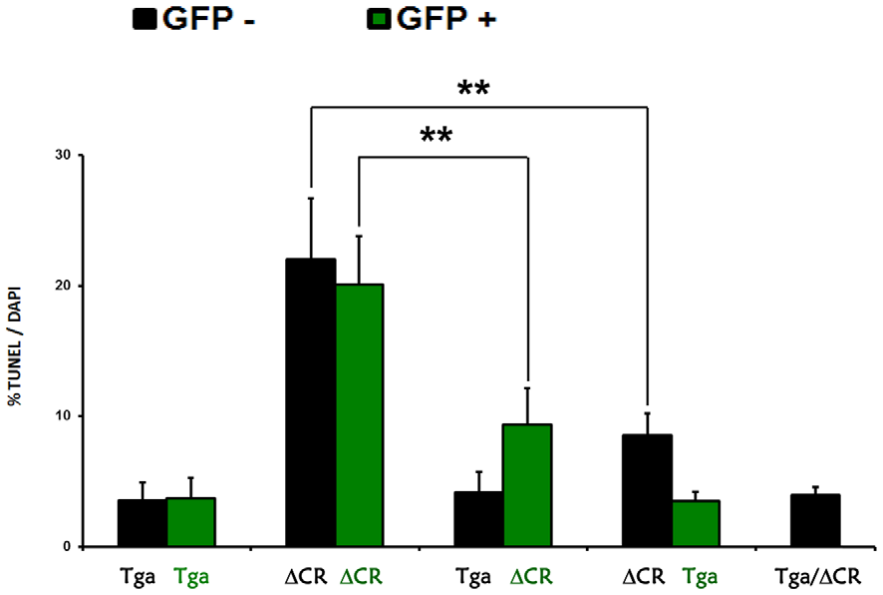
WT PrP rescuing activity can be exerted *in trans*. Mixed cultures of GFP-negative or GFP-positive NSCs from of Tg*a* or ΔCR mice were treated and analyzed as described in Figure 8. The bar graph shows the number of TUNEL-positive cells, expressed as a percentage of the number of DAPI-stained cells, as determined in 5 fields for each sample group. Bars show means ± SEM (n = 4 independent experiments). The number of TUNEL-positive ΔCR cells, either GFP-positive or GFP-negative, was significantly reduced when these cells were co-cultured with WT PrP-expressing Tg*a* cells (** p<0.01).

First we mixed green and black Tg*a* NSCs (Figure 9). As expected, we found that only a small percentage (<3%) of these cells, either green or black, were TUNEL-positive after exposure to Zeocin.

Then, we mixed black Tg*a* and green ΔCR neurospheres, and measured the percentage of TUNEL-positive cells after treatment with Zeocin. As positive control for the rescue, we treated with Zeocin black NSCs derived from doubly Tg mice co-expressing ΔCR PrP and the Tg*a* transgene (Tg*a*/ΔCR NSCs). As expected, we found only few (>5%) TUNEL-positive nuclei in both black Tg*a* and Tg*a*/ΔCR NSCs. Surprisingly, we detected a significantly reduced number of TUNEL-positive nuclei in green ΔCR NSCs co-cultured with black Tg*a* NSCs, as compared to control green ΔCR cells cultured alone. This observation was confirmed by mixing green Tg*a* NSCs with black ΔCR NSCs. Also in this case, we detected a much lower percentage of TUNEL-positive nuclei in black ΔCR NSCs co-cultured with green Tg*a* NSCs, as compared to black ΔCR cultured alone. These results indicated that WT PrP can exert its rescuing effect *in trans*. Interestingly, we found that the number of TUNEL-positive nuclei in ΔCR NSCs co-cultured with Tg*a*20 NSCs was still significantly higher than in Tg*a*/ΔCR NSCs co-expressing the WT and mutant proteins in the same cells (Figure 9). This result suggests that the trans-rescuing effect of Tg*a* NSCs toward ΔCR cells was not as efficient as the cis-rescuing effect.

### Cis-toxicity of ΔCR and trans-rescuing activity of WT PrP are both detectable in HEK293 cells

**Figure 10:**
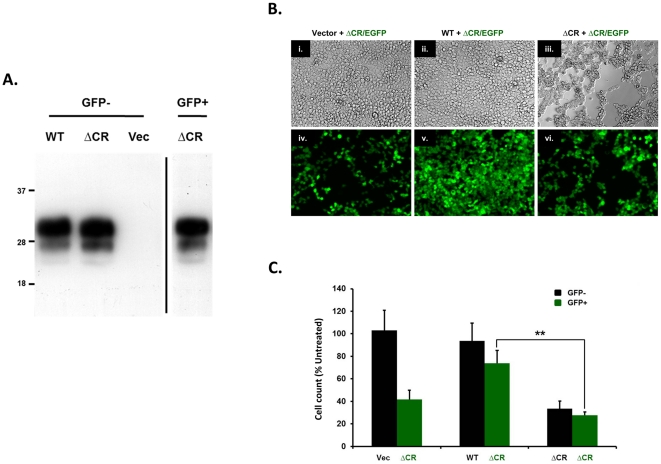
Cis toxicity of ΔCR and trans rescuing activity of WT PrP are detectable in HEK293 cells. (**A**) Equivalent amounts of total protein from lysates of HEK293 cells stably transfected with the empty vector (Vec), or with vectors encoding for WT or ΔCR PrPs, were analyzed by Western bloting using 6D11 antibody to detect PrP. A homogenous population of ΔCR cells stably expressing GFP (GFP+) was obtained by lentiviral transduction and FACS sorting. Other cells were not transduced with virus (GFP−) (**B**) GFP+ ΔCR cells were mixed with GFP− cells expressing Vector, WT or ΔCR PrP. Co-cultures were treated for 48 hrs with Zeocin (500 µg/ml), and imaged by detecting GFP fluorescence (i.–iii.), or by phase contrast (iv.–vi.). (**C**) The bar graph shows the total number of cells after Zeocin treatment, expressed as a percentage of the number of untreated cells, as determined in 8 fields for each sample group. Bars show means ± SEM (n = 4 independent experiments). GFP+ ΔCR-expressing cells are significantly more susceptible to the Zeocin than co-cultured Vector cells (p<0.001). Moreover, the number of surviving GFP+ ΔCR cells is significantly increased when these cells are co-cultured with GFP− cells expressing WT PrP, but not with GFP− cells expressing ΔCR PrP (** p<0.01).

We sought to test whether our observations in differentiated NSCs also applied to non-CNS derived cell types. We turned to HEK293 cells, which we have used extensively as a cell model for the DBCA [15], [16]. In order to mimic the approach described for NSCs, we transduced HEK293 cells stably expressing ΔCR PrP with a recombinant lentivirus encoding for cytoplasmic GFP. A homogenous population of ΔCR cells (∼99%) highly expressing GFP was then obtained by fluorescence activated cell sorting (FACS) (data not shown). Importantly, these cells maintained the same ΔCR PrP expression level as the original clone (Figure 10A).

In order to test the cis-hypersensitizing effect of ΔCR PrP and the trans-rescuing activity of WT PrP, green ΔCR cells were mixed in a 1∶1 ratio with black (non-transduced) cells expressing either empty vector, WT or ΔCR PrP, and then treated with Zeocin for 48 h (Figure 10B). As a read-out, we counted the total amount of cells, either green or black (Figure 10C). As expected, when green and black ΔCR cells were mixed, the treatment with Zeocin diminished the number of both green and black cells to about 30% compared to untreated cells. In contrast, when green ΔCR cells were mixed with vector-transfected cells, Zeocin diminished only the number of green ΔCR cells, while the number of vector cells was unaffected. This result confirmed that ΔCR-dependent hypersensitivity to Zeocin is cell autonomous. Morevoer, we detected a significant rescue of green ΔCR cells when these were co-cultured with WT-expressing cells. This confirmed the ability of WT PrP to rescue ΔCR-dependent hypersensitivity *in trans*.

Collectively, these results indicate that the cis-toxicity of ΔCR PrP and the trans-rescuing activity of WT PrP can both be detected in a non-CNS derived, transformed cell type (HEK293 cells).

## Discussion

Expression of ΔCR PrP sensitizes several different cell lines to certain cationic antibiotics, including Zeocin, G418 and hygromicin, an effect that can be quantitated by the DBCA [16]. This drug-sensitizing effect is directly correlated with the ability of ΔCR PrP, and related mutant PrPs, to induce ion channels in the cell membrane, and with the neurotoxicity of these mutants in transgenic mice [11]. Importantly, co-expression of WT PrP suppresses both the drug hypersensitivity and ion channel activity of ΔCR PrP in cultured cells [15], analogous to the ability of WT PrP to reverse the neurodegenerative phenotype of Tg(ΔCR) mice [11]. Thus, the DBCA measures a physiologically relevant, functional activity of PrP.

Here, we have used a novel format of the DBCA to further characterize the drug-hypersensitizing effect of ΔCR PrP and the rescuing activity of WT PrP in differentiated NSCs. We found that ΔCR induces hypersensitivity to drugs in a cell-autonomous fashion (i.e., *in cis*), and that this effect is suppressed by WT PrP supplied from adjacent cells (i.e., *in trans*). These results suggest that two topologically different states of PrP are responsible for the cytotoxic and cytoprotective activities of the protein, with the first acting on the same cell to which PrP is attached, and the second directed toward neighboring cells.

### The toxicity ΔCR PrP is cell-autonomous

By co-culturing NSCs that express ΔCR PrP with those that lack PrP expression, we were able to assess whether the toxic activity of ΔCR PrP is cell-autonomous. We found that, while NSCs expressing ΔCR PrP were killed by Zeocin, adjacent cells lacking PrP were not. These results suggest that ΔCR PrP acts cell-autonomously to confer Zeocin sensitivity. Our results argue against the possibility that soluble forms of ΔCR PrP, or other toxic molecules, are released from cells and act on adjacent cells, or that membrane-bound ΔCR PrP exerts toxic effects on closely opposed cells. In our experimental system, NSCs with and without PrP were co-cultured and then differentiated into the three major cell types (neurons, astrocytes, and oligodenrocytes), so that ΔCR PrP-expressing cells were closely interdigitated with *Prn-p* ^0/0^ cells. However, our results do not rule out possible trans-effects of ΔCR PrP, if these were significantly less potent than the cis-effects.

Our data are consistent with several previous studies in transgenic mice indirectly suggesting that ΔCR PrP, as well as other PrP deletion mutants, exert their toxicity *in cis*. In addition to ΔCR PrP, several other PrP mutants carrying deletions spanning the central region, including Δ32–121, Δ32–134 and Δ94–134, have been shown to induce progressive neurodegeneration in mice, characterized by loss of cerebellar granular neurons, astrogliosis, and white matter vacuolization [20], [21]. The expression pattern of these mutant PrPs in the brain is similar to that of endogenous PrP^C^, with the exception that cerebellar Purkinje cells do not express the protein because the transgenic vector lacks a Purkinje cell-specific enhancer element. Purkinje cells, in contrast to granule neurons, do not degenerate in these Tg lines [11], [20], [21]. This observation indicates that, as is the case for ΔCR PrP, the toxicity of the other PrP deletion mutants is also cell autonomous, and is not transferred to neighboring cells.

### WT PrP displays a trans-rescuing effect, which may reflect a physiological activity of the protein

To assess the trans-rescuing activity of WT PrP, we co-cultured NSCs expressing ΔCR PrP with NSCs expressing WT PrP. We observed that WT PrP acted *in trans* to suppress the Zeocin hypersensitivity of nearby cells expressing ΔCR PrP. Interestingly, this rescuing effect was only partial, since Zeocin sensitivity was not reduced to the level observed when the Tg*a*20 (WT PrP) and ΔCR PrP transgenes were expressed in the same cell. One possible explanation for this difference is that WT PrP exerts its rescuing activity both *in cis* and *in trans*, with the first effect being stronger than the second effect. An alternative possibility is that the trans-rescuing effect exerted by WT PrP requires physical contact between ΔCR PrP and Tg*a*20 NSCs. In this case, the absence of complete rescue could indicate that a small percentage of ΔCR cells are not sufficiently close to Tg*a* NSCs in the mixed culture.

The trans-rescuing effect of WT PrP observed here is consistent with several previous observations. For example, a trans-rescuing effect of WT PrP has also been observed in Tg(F35) mice which express Δ32–134 PrP. Expression of a soluble form of PrP lacking the GPI anchor, or expression of WT PrP restricted to astrocytes, counteracted neuronal loss and white matter pathology, and prolonged survival of Tg(F35) mice [22]. Similar results were obtained in Tg mice expressing Doppel (Dpl), a PrP paralog that is structurally homologous to Δ32–134 PrP and that induces a neurodegenerative phenotype when ectopically expressed in the brain [23]. In this case, degeneration of Purkinje cells expressing Dpl was rescued by WT PrP expression in surrounding neurons and glia [24], [25]. Collectively, these results indicate that neurotoxicity of several PrP mutants and Dpl can be rescued *in trans* by WT PrP.

The trans-rescuing activity of WT PrP may reflect a physiologic activity of the molecule. Several pieces of evidence indicate that PrP^C^ can function in a trans-cellular fashion in a variety of physiological contexts. For example, PrP^C^ can act as a cellular adhesion molecule, participating in neuronal survival, neurite outgrowth, and synaptic function [7], [9], [26]. Another recent study found that several PrP knockout mouse strains exhibited a chronic demyelinating polyneuropathy when PrP^C^ was depleted from neurons but not Schwann cells [27]. Interestingly, this pathology was rescued when neuronal expression of PrP^C^ was restored, suggesting that axonal PrP^C^ exerts a role in long-term maintenance of myelin by interacting *in trans* with receptors or other molecules on the surface of Schwann cells. Taken together, these data indicate that PrP^C^ acts in a trans-cellular fashion in a variety of important physiological processes. Therefore, the trans-rescuing effect of WT PrP toward ΔCR-expressing cells observed in this study could directly reflect a biological activity of the molecule. Further characterization of the molecular aspects of WT PrP trans-rescuing activity, and the identification of PrP domains involved in this phenomenon, will likely provide fundamental insights into the normal function of the protein.

### Models to explain the cell-autonomous toxicity of ΔCR PrP and the trans-rescuing activity of WT PrP

In addition to its ability to hypersensitize cells to several cationic antibiotics, ΔCR PrP induces spontaneous inward currents in cells that can be recorded by patch-clamping techniques [13]. There is a close correlation between the current-inducing activity of different PrP mutants and their drug-sensitizing activity, suggesting that the two processes are mechanistically related, perhaps because the cationic antibiotics pass through the PrP-related channels. Characterization of the biophysical properties of the ΔCR-induced currents indicates that they are produced by relatively non-selective, cation-permeable channels or pores in the cell membrane. ΔCR PrP-induced currents have been observed in cells of both neuronal and non-neuronal origin from a variety of species, ranging from insects to mammals. Therefore, we previously hypothesized that ΔCR PrP regulates the activity of an endogenous channel that is extremely well conserved and widely expressed, or more likely, that the ΔCR PrP molecule itself forms channels independent of other cellular proteins [13]. Both possibilities are consistent with the data presented here, which show that ΔCR-induced hypersensitivity to antibiotics is exerted in a cell autonomous fashion, as would be expected for a protein that either forms or modulates a channel in the cell membrane.

**Figure 11:**
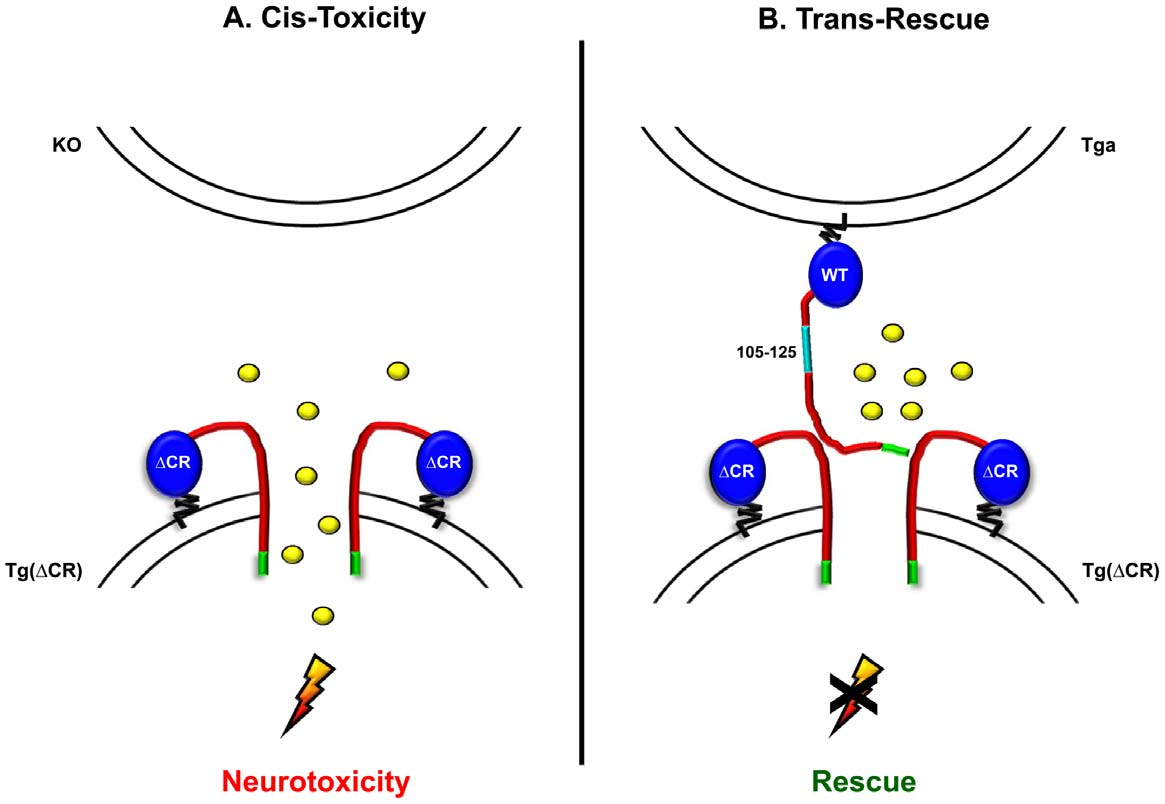
Models for ΔCR PrP cis-toxicity and WT PrP trans-rescue. (**A**) Data from previous reports suggest that ΔCR PrP forms a channel or pore in the plasma membrane, which allows cationic molecules (illustrated as yellow balls) such as the antibiotics used in the DBCA, to enter the cell. (**B**) WT PrP on the surface of one cell exerts its protective effect *in trans* by silencing the channel on neighboring cells. A similar effect could be produced by PrP molecules released into the medium or physically transferred from neighboring cells (not shown). The N-terminal polybasic domain (residues 23–31, indicated in green) plays an important role in both the cis-toxicity of ΔCR PrP and the trans-rescuing effect of WT PrP.

Recent studies have demonstrated that the N-terminal polybasic domain (residues 23–31) is essential for the neurotoxicity, ion channel activity, and drug-sensitizing capability of ΔCR PrP and other N-terminally deleted PrP mutants [14], [28]. This region has also been shown to function as a protein transduction domain capable of ferrying polypeptides across the plasma membrane [29], [30]. We hypothesize that residues 23–31 contribute to the cis-activity of ΔCR PrP by allowing the polypeptide to insert into the plasma membrane and form a pore (Figure 11A). In this scenario, our data would imply the existence of a molecular or structural constraint which prevents the PrP polypeptide from penetrating the plasma membrane of surrounding cells and exerting its toxic effect *in trans*.

What is the mechanism for the trans-rescuing effect of WT PrP? We have previously shown that ΔCR-dependent currents are silenced by co-expression of WT PrP in the same cell [13]. One possible explanation for this phenomenon is that WT PrP closes the pore formed by ΔCR PrP, perhaps by physically interacting with the mutant protein (Figure 11B). The data presented here suggest that this function can be performed by WT PrP supplied from an adjacent cell, either attached to the membrane (Fig. 11B), or in a soluble form (not shown). It is known that PrP^C^ is spontaneously released from cells by the action of endogenous phospholipases, or by proteases that generate N-terminal fragments [31], [32], [33]. In addition, intercellular transfer of GPI-anchored PrP^C^ has been previously reported [34]. Interestingly, we have shown that deletion of residues 23–31 greatly diminishes the rescuing activity of WT PrP both in cell culture assays and transgenic mice [28], [35]. Therefore, in addition to playing a role in the toxicity of ΔCR and other mutant forms of PrP, residues 23–31 are also important for the neuroprotective activity of WT PrP.

### Does prion toxicity involve a cell-autonomous activity of PrP^C^?

Several lines of evidence suggest that prion-induced neurodegeneration is directly dependent on the presence of PrP^C^ on the neuronal surface [5], [36]. For example, genetic deletion of neuronal PrP^C^ in infected mice reverses some aspects of neuropathology and prolongs survival, despite the continued presence of PrP^Sc^ [5], [6]. Moreover, the absence of the glycolipid membrane anchor on PrP^C^, which results in secretion of the protein into the extracellular space, causes dramatic changes in the characteristics of scrapie-induced illness in mice [37]. Collectively, these observations indicate that a normal, cell-autonomous activity of PrP^C^ mediates PrP^Sc^ toxicity. This conclusion underlines an interesting parallel between the cis-toxicity of ΔCR PrP and the neurotoxicity of PrP^Sc^. What is the explanation for this correlation? In one possible scenario, binding of PrP^Sc^ to neuronal PrP^C^ could force the latter to insert its N-terminus into the lipid bilayer and produce a ΔCR-like channel, resulting in the generation of abnormal ionic currents at the plasma membrane, and subsequent neurotoxicity [8]. Further studies will be necessary to determine whether abnormal, PrP-related channel activity occurs in cells infected with prions.

## Materials and Methods

### Ethics Statement

This study was carried out in strict accordance with the recommendations in the Guide for the Care and Use of Laboratory Animals of the National Institutes of Health. The protocol was approved by the Boston University Institutional Animal Care and Use Committee (Permit Number: AN-14997).

### Mice

Generation of Tg*a*20^+/+^/*Prn-p^0/0^* (Tg*a*) and Tg(ΔCR)/*Prn-p^0/0^*(ΔCR) mice has been described previously. Tg(*ACTB-EGFP/Prn-p^0/0^*) mice were obtained by crossing C57BL/6-Tg(ACTB-EGFP) mice (The Jackson Laboratory, Bar Harbor, Maine) twice with *Prn-p^0/0^* mice on the C56BL6 background (EMMA, Munich, Germany). In order to obtain NSCs of the different genotypes, either green or black, Tg(ΔCR) mice expressing one copy of the Tg*a*20 transgene on the *Prn-p^0/0^* background [ΔCR^+/−^/Tg*a*20^+/−^/*Prn-p^0/0^*] were mated to Tg(*ACTB-EGFP^+/−^/Prn-p^0/0^*) mice. E13.5 mouse embryos were genotyped by PCR analysis of limb DNA. DNA was prepared using the Puregene DNA Isolation Kit (Gentra Systems, Minneapolis, MN).

### NSCs

Neural stem cells were obtained and cultured following a procedure described previously [19], with minor modifications. Brains dissected from E13.5 mouse embryos were triturated in 5 ml of NeuroCult NSC basal medium containing NeuroCult NSC proliferation supplement (StemCell Technologies,Vancouver, BC) along with 20 ng/ml EGF. Once formed, neurospheres were differentiated by pipetting a 0.1–1 ml suspension (containing approximately 30–40 mature neurospheres) into each well of an 8-well chamber slide (Ibidi GmbH, München, Germany) containing NeuroCult NSC basal medium with NeuroCult NSC differentiation supplement (StemCell Technologies) along with 10 µg/ml retinoic acid.

The DBCA was performed as described previously [16], with minor modifications. Briefly, NSCs differentiated for 10 days were treated with 500 µg/ml of Zeocin for 24 hrs, and stained by TUNEL or with DAPI (see below) to assess cell death.

### Western blots

Ten-percent (w/v) homogenates of mouse brain were prepared using a glass/Teflon apparatus (10 strokes at 1,000 rpm) in ice-cold Triton-DOC buffer (0.5% Triton-X-100, 0.5% sodium deoxycholate, 150 mM NaCl, 50 mM Tris-HCl, pH 7.5), plus protease inhibitors. The same buffer was also used to lyse NSCs. Brain or cell lysates were centrifuged at 900 *xg* for 10 min to remove debris prior to analysis by SDS-PAGE. In some cases, proteins were enzymatically deglycosylated with PNGase F according to the manufacturer's directions (New England Biolabs, Beverly, MA). Samples were then diluted 1∶1 in 2× Laemli sample buffer (2% SDS, 10% glycerol, 100 mM Tris-HCl pH 6.8, 0.002% bromophenol blue, 100 mM DTT), heated at 95°C for 10 min, then analyzed by sodium dodecyl sulfate polyacrylamide gel electrophoresis (SDS-PAGE). Proteins were electrophoretically transferred to polyvinylidene fluoride (PVDF) membranes, and membranes were blocked for 10 min in 5% (w/v) non-fat dry milk in Tris-buffered saline containing 0.005% Tween 20. After incubation with appropriate primary and secondary antibodies, signals were revealed using enhanced chemiluminescence (Amersham Biosciences, Uppsala, Sweden), and visualized by a Biorad XRS Chemidoc image scanner (Biorad, Hercules, CA).

### Antibodies

Monoclonal anti-PrP antibody 6D11 (provided by Richard Kascsak, Institute for Basic Research in Developmental Disabilities, Staten Island, NY, USA), rabbit anti-glial fibrillary acidic protein (GFAP) (Dako, Carpinteria, CA), anti-nestin (Clone Rat 401, StemCell Technologies) and anti-MAP-2 were all diluted 1∶2,000 for Western blot and 1∶1,000 for immunofluorescence.

### Immunofluorescence staining

NSCs were differentiated in 8-well laminin-coated chamber slides for 7–10 days. Cells were fixed with 4% paraformaldehyde in PBS for 20 min, permeabilized with 0.5% Triton-X-100 in PBS for 10 min, treated with blocking buffer (PBS+ 2% goat serum) for 30 min, then stained with appropriate primary and secondary antibodies (1∶1,000) in blocking buffer for 1 h. Nuclei were counterstained with 1 µg/ml DAPI (Sigma Aldrich) for 5 min. For terminal deoxynucleotidyl transferase dUTP nick end labeling (TUNEL), cells plated on glass coverslips were fixed with 4% paraformaldehyde for 20 min, rinsed twice with PBS, permeabilized with 0.5% Triton X-100 in PBS for 10 min, and then stained using a TMR Red In Situ Cell Death Detection Kit, according to manufacturers' directions (Roche Applied Science, Indianapolis, IN). Cell nuclei were counterstained with DAPI. Glass slides were mounted with Gel/Mount (Biomeda, Foster City, CA) and imaged on a Nikon TE2000E2 inverted fluorescence microscope equipped with a CCD camera. Images were processed using MetaMorph software (Molecular Devices, Sunnyvale, CA). The number of TUNEL-positive cells, expressed as a percentage of DAPI-positive cells, was determined in at least five fields for each sample group.

### Time-lapse microscopy

Images were collected and analyzed with a Cell-R imaging station (Olympus, Japan) coupled to an inverted microscope (IX 81, Olympus) equipped with an incubator to maintain constant temperature (37°C) and CO_2_ (5%) values. Phase contrast and fluorescence (GFP) images were acquired with a high-resolution camera (ORCA, Hamamatsu, Japan) at 20× magnification. Fluorescence images were acquired using an excitation filter of 450–480 nm, a dichroic mirror of 500 nm, and an emission filter of 515 nm (Olympus). Five different frames, randomly sampled, were analyzed. Images were acquired at 10 min intervals for a total of 72 hours.
